# Selling a Practice

**Published:** 1847-08

**Authors:** 


### ARTICLE VI.

### SELLING A PRACTICE.

Fashions in the mode of business amongst medical men, as in drapery amongst the people, varies in different districts of the country.

In New England physicians sell their practice; as concluded from the following advertisement in the Boston Med. and Surg. Jour., and other evidence:

"An elderly physician, in Worcester county, wishing to relinquish business, offers his practice, and a small amount of real estate, on liberal terms," &c.

In the western country, physicians would find *great difficulty*, if not in making out a bill of sale, at least in giving a clear and *indisputable* title in the transfer of their practice.

